# Hidden Potential: An Exploratory Study of the Prevalence of Twice-Exceptional Students in Saudi Arabia

**DOI:** 10.3390/jintelligence14050083

**Published:** 2026-05-10

**Authors:** Norah Almulhim, Abdullah Aljughaiman, Fahad Alnaim, Abdulrahman Alsayed, Sarah Alfawzan, Abdulhamid Alarfaj

**Affiliations:** Special Education Department, King Faisal University, Al-Ahsa 36362, Saudi Arabia; faalnaim@kfu.edu.sa (F.A.); aalsayed@kfu.edu.sa (A.A.); salfozan@kfu.edu.sa (S.A.); abdarfaj@kfu.edu.sa (A.A.)

**Keywords:** twice exceptional, prevalence, identification, learning disabilities, giftedness

## Abstract

Although twice-exceptional students are receiving more attention than before, the topic is still under-researched. This poses a major problem for this group, as a comprehensive educational policy that meets its needs can only be developed based on realistic estimates. Therefore, this study aimed to explore the prevalence of twice-exceptional students in Saudi Arabia. It also sought to investigate whether prevalence rates vary according to gender, age, and academic subject. The sample included 6875 students ranging from second to twelfth grade. These students were screened via a two-step process. First, students with potential learning disabilities based on a discrepancy of more than 1.5 standard deviations between their academic achievement in two subjects (e.g., between Arabic and math) were flagged for further assessment. Second, a giftedness test was administered to these students to screen for gifted students. The first step suggested that 23.6% of students showed indicators of learning disabilities. The intelligence test in the second stage indicated that 24.5% of students with potential disabilities may be gifted. The preliminary analysis suggests that approximately 5.9% of the sampled student population may be twice-exceptional, which represents a substantial number of underserved students. This suggests the potential value of developing educational policies and practices to meet the needs of this population, although broader replication is needed before definitive policy recommendations can be made.

## 1. Introduction

The topic of twice-exceptional students in general and gifted students with learning disabilities in particular has recently gained increasing recognition in the literature on gifted education. However, deeper knowledge and awareness of this concept remain limited within educational and psychological communities, and professionals often lack the experience essential to effectively work with this group of learners (Foley-Nicpon et al., 2013). This has led to a severe lack of provision of appropriate educational services in both Western and Arab contexts. Bakhiet and Essa (2012) noted a dearth of programs addressing the identification, diagnosis, and right approach to education for this group in the Arab world, despite the multitude of classroom obstacles faced by this group, given their diverse educational, motivational, and behavioral characteristics (Hughes, 2011). Therefore, this calls for an urgent measure to offer educational support to nurture their talents while also addressing their challenges.

The term “twice exceptional” in this paper refers to the coexistence of giftedness and one or more disabilities. Furthermore, it highlights this study’s focus on the intersection of giftedness and learning disabilities, which represents a prominent group within the twice-exceptional population. Twice-exceptionality can be understood as a complex interaction between giftedness and coexisting disabilities (Rizzo et al., 2025), whereby students may exhibit characteristics associated with giftedness alongside disability-related traits across cognitive, behavioral, and social–emotional domains (King, 2022). Cognitively, they may demonstrate advanced comprehension, creativity, and abstract thinking (Alsamani et al., 2023), while also scoring low on academic performance or experiencing difficulties with attention and concentration (Alsamani et al., 2023; Cormier, 2025). Research highlights the occurrence of both heightened and lower social and emotional maturity relative to their chronological age and strong problem-solving abilities in a few cases (Lovecky, 2023; Alsamani et al., 2023). This uneven profile becomes even more pronounced with the co-occurrence of high intellectual ability and significant areas of weakness, which may lead to misidentification as learning disabilities, attention-deficit/hyperactivity disorder, or autism (Silverman, 2024). In addition, giftedness may mask the severity of difficulties, making the disability less visible and obscuring students’ actual needs, as they may appear capable of achieving acceptable academic outcomes despite performing below their true potential (Baum et al., 2017).

The knowledge of learning disabilities is particularly important within the broader field of twice-exceptionality, attributable to the highly complex and often inconsistent approaches to recognizing gifted students with learning disabilities (Ozturk & Tan, 2025; Beumann et al., 2025). In general, students with learning disabilities face significant and persistent difficulties in one or more academic areas, such as reading, writing, or mathematics, despite access to appropriate learning opportunities. However, understanding these difficulties is not straightforward because related judgments are shaped by different models, policy interpretations, and assessment procedures (Fletcher & Miciak, 2024; Harris et al., 2024). In the Saudi context, learning disabilities remain conceptually complex and are understood through multiple perspectives rather than through a single fixed definition. They are generally linked to difficulties in cognitive processes such as perception, memory, and language, which may interfere with academic achievement (Alnaim & Sakız, 2025). Within Saudi educational practice, the identification of students with learning disabilities has largely been informed by discrepancies between mental ability and academic achievement across domains such as reading, writing, mathematics, listening, and verbal expression, alongside consideration of related cognitive processes, such as memory, attention, thinking, and perception. This process also applies exclusionary criteria to distinguish learning disabilities from other disabilities and from difficulties that are primarily attributable to economic, social, or unsuitable learning conditions (Saudi Ministry of Education, 2021).

This complexity becomes even more pronounced among gifted students whose strengths may mask academic difficulties, with their gifted potential making them vulnerable to under-recognition and inconsistent educational interventions (Beumann et al., 2025). This challenge is also reflected in the broader literature on twice-exceptionality, where discrepancies between performance indicators have frequently been recruited to explore the profiles of twice-exceptional students. Ozturk and Tan (2025) highlight that some studies have examined discrepancies between intelligence and academic achievement measures, while others have considered inconsistencies across subscale or subindex scores within formal assessment batteries. Taken together, these patterns suggest that uneven academic performance may provide a useful exploratory signal in school-based screening of potentially gifted students with learning disabilities, particularly in large-scale contexts. In this sense, such patterns are better understood as preliminary indicators that can inform exploratory investigation rather than serving as definitive evidence for formal identification.

The broader context of learning disabilities also adds to the existing complexity of examining this population. Estimates of learning disabilities vary considerably across reports and studies, and this variation is influenced by differences in sample characteristics, inclusion criteria, and diagnostic approaches (Agrawal et al., 2019). Differences across countries and shifts in diagnostic frameworks have further contributed to this variability, including changes associated with the classification of learning difficulties within neurodevelopmental disorders and the evolving role of discrepancy-based criteria in diagnostic practice (Misciagna, 2020). Recent evidence also suggests that prevalence estimates continue to vary across contexts. For example, Li et al. (2023) reported prevalence estimates of 7.7% and 7.86% among children and adolescents in the United States based on national survey data, while other studies have reported approximately 6% in Brazil (Fortes et al., 2016) and around 8% in India (Scaria et al., 2023). In the Arab world, the available evidence is still sparse and often rests on relatively small samples or narrow academic domains, particularly reading difficulties. In Saudi Arabia, accurate national figures are not yet available, and current estimates are largely inferred from service provision, with approximately 7% of school students reported to receive learning-disabilities services (Abu Nayan, 2021).

A similar pattern of variability appears in the literature on gifted students with learning disabilities. The available evidence suggests that estimates for this group differ substantially across studies and contexts, partly because of differences in how giftedness and learning disabilities are identified and combined within the same student. In the United States, estimates from studies have placed this group between 300,000 and 360,000 students, or about 0.5% of children under the age of 18 (Baum et al., 2017; National Association for Gifted Children, 2009). Other estimates have highlighted that 2% to 7% of students are placed under special education settings (Trail, 2011); values from 2% to 5% of total students have been found in some administrative estimates; and values of up to 9.1% of the total student population have been obtained when academic talent is considered more broadly (Barnard-Brak et al., 2015). Additional work has emphasized that precise figures remain difficult to establish since many of these students may go unrecognized, underestimated, or effectively “disappear” within conventional identification systems (Silverman, 2018). More recent estimates in the United States have placed gifted students with learning disabilities between 1.1% and 4.3% of students (Maddocks, 2020), while other international estimates have also varied, including 10% of talented individuals in Australia (Ronksley-Pavia, 2020). In Saudi Arabia, research on this group remains limited. One of the few available studies is that of Bakhiet and Essa (2012), who examined gifted children with learning difficulties within a learning difficulties program in Riyadh and reported a prevalence rate of 3.3%. Their findings also highlighted the importance of improving evaluation and diagnosis procedures to reduce misclassification and ensure appropriate services for this group. Overall, the Saudi evidence remains restricted in scope and scale, leaving a significant gap in the local literature and reinforcing the need for broader exploratory work on gifted students with learning difficulties in the Saudi context.

To address this gap, this study seeks to provide an exploratory estimate of the prevalence of twice-exceptional students in Saudi Arabia through a large-scale approach. This study is significant as it may provide deeper insights into improving the understanding of this group, which may inform educational planning, teacher preparation, program development, and support services, while also contributing to the limited Arab literature on twice-exceptionality. This study addresses two main questions: first, what is the estimated prevalence rate of twice-exceptional students in Saudi Arabia, and to what extent does this estimated prevalence vary by gender, age group, and program type? Second, to what extent does the prevalence of learning disabilities vary across academic subjects?

## 2. Materials and Methods

### 2.1. The Participants

The participants were 6875 students from first to 12th grade from four schools in Riyadh. The students ranged in age from 8 to 19 years (M = 12.95; SD = 2.59), of whom 40% were female, 60% were male, and 74% were from the local program schools, while the rest were from the international program schools. The number of classes ranged from 10 to 36 classes in each school year.

### 2.2. Measures

#### 2.2.1. School Grades

The instrument used to screen for students showing indicators of learning disabilities was their grades in language (which, in this study, comprised the Arabic subject), mathematics, and science. These three subjects were selected because they represent the core academic areas assessed at all levels of the Saudi national curriculum (Saudi Ministry of Education, 2021) and are the primary subjects in which learning disabilities most commonly occur: language, mathematics, and written/scientific expression (American Psychiatric Association, 2022). Although Arabic was included as a screening subject for students in the international program, it is a compulsory core subject for all students from the first year, regardless of program type. This makes a mismatch between the curriculum and language an unlikely explanation for the discrepancies observed.

The Ibn Akhladon Educational Group used an achievement standardized test for these subjects for all students in their schools. Each school year, the school’s administration implements these tests to evaluate students’ learning and the acquisition of basic skills related to the subjects. The items in the test assess the grade-level expectations for each subject and grade level. Students’ grades can range from 0 (the lowest possible grade) to 100 (the highest possible grade). Students’ grades were supplied by the school administrations. Cronbach’s alpha for all grade levels ranges from 0.72 to 0.83 for Arabic, from 0.78 to 0.83 for mathematics, and from 0.80 to 0.84 for science.

#### 2.2.2. Students Showing Indicators of Learning Disabilities

Students who had evidence of academic impairment based on a discrepancy of >1.5 standard deviations between two of the three academic subjects (e.g., between Arabic and mathematics) were coded as 1, while students without indicators of learning disabilities were coded as 0. This criterion was used as an initial screening process to identify students with significant and consistent discrepancies in their academic performance across core subjects.

#### 2.2.3. Gender

Students’ gender was reported by the school administrations. A male student was coded as 1, while a female student was coded as 0.

#### 2.2.4. School Level

The reported grade levels of the students were categorized and coded as follows: 1 (includes students from first to sixth grade; primary school), 2 (includes students from seventh to ninth grade; intermediate school), and 3 (includes students from tenth to twelfth grade; high school).

#### 2.2.5. Program Type

A program in which the curriculum for all subjects was taught in Arabic was called a local program and was coded as 0. A program in which the curriculum for all subjects was taught in English (except for the Arabic subject) was called an international program and was coded as 1.

#### 2.2.6. Successful Intelligence Test

The successful IQ test measures a student’s intellectual abilities and consists of three main sections: verbal intelligence, numerical intelligence, and non-verbal intelligence. The Successful Intelligence Test used in this study is based on Sternberg’s theory of successful intelligence and is derived from the Aurora Battery (Chart et al., 2008). In Saudi Arabia, the battery was translated, adapted, and standardized by the National Research Centre of Giftedness and Creativity at King Faisal University on a large national sample of 7800 students drawn from different regions of the Kingdom. In addition, previous Saudi psychometric work supported the construct validity and factor structure of the battery, and large-scale Saudi studies have also used the Aurora framework successfully (A. M. Aljughaiman & Ayoub, 2012; Hein et al., 2015; Ayoub & Aljughaiman, 2016). Students were coded as gifted if they scored ≥120 on the successful intelligence test.

### 2.3. Procedure

Ethical approval was obtained, and a sampling design was selected before data collection. First, approval for the data collection procedure and the instruments of this study was sought from the Ethics Committee of King Faisal University. Then, schools were contacted to gain access to their students and to gather their demographic information. Because of the large number of schools (568 schools) in Riyadh (the capital city of Saudi Arabia), it was not possible to include all schools. Consequently, convenience non-random sampling was utilized. With four schools in different parts of Riyadh, Ibn Akhladon Educational Groups were chosen. Each of these schools has two sections: the first is local and all subjects are taught in Arabic; the second is international, and all subjects are taught in English.

This study was conducted in two stages: in the first stage, students’ grades in the main schools’ subjects (language, mathematics, and science) were collected to identify students who had a discrepancy between two academic subjects (e.g., between language and mathematics) as a criterion for the presence of indicators of learning disabilities. Then, in the next stage, the successful intelligence test was administered to the students identified in the first stage. At this stage, consent was obtained from schools and parents before the successful intelligence test was administered.

### 2.4. Plan of Analysis

This study’s main objective was to calculate the prevalence of twice-exceptional students in Saudi Arabia, specifically gifted students with learning disabilities. Therefore, in the first stage, we identified students with indicators of learning disabilities by screening for evidence of academic impairment based on a discrepancy of >1.5 standard deviations between two of the main academic subjects (i.e., between Arabic and mathematics, Arabic and science, or mathematics and science, and vice versa). In the next stage, we administered the successful intelligence test to the identified students with indicators of learning disabilities from the previous stage. Then, we used descriptive statistics to estimate the prevalence of gifted students among students with indicators of learning disabilities, disaggregating the data by school level, gender, and school program. For comparative analysis, we used chi-squared tests to compare prevalence rates among different groups (e.g., boys vs. girls, international vs. local schools, primary vs. secondary vs. high school, and learning disabilities in specific academic subjects). To account for the increased risk of type I errors across multiple comparisons, a Bonferroni correction was applied, adjusting the significance threshold to α = 0.0125. All statistical analyses were conducted using IBM SPSS Statistics (Version 26; IBM Corp., Armonk, NY, USA).

## 3. Results

### 3.1. Preliminary Analyses

Before the main analysis, we examined the psychometric properties of the study variables. The descriptive statistics, including means, standard deviations, and reliability coefficients for students’ grades in Arabic, mathematics, and science, as well as for the successful intelligence test, are presented in Table 1.

### 3.2. Prevalence of Gifted Students with Learning Disabilities

**Figure 1:**
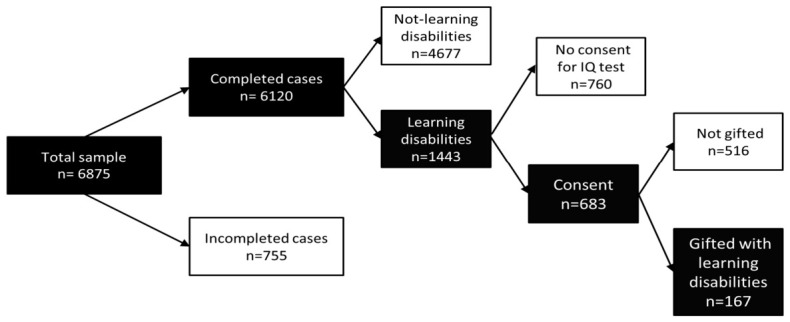
The study flowchart.

Of the 6875 students in the Ibn Akhladon educational groups included in this study, 6120 (89%) cases were included because they were completed with respect to the information of at least two of the selected subjects (see Figure 1 for the study flowchart). The general formulae (Arabic > math), (science > math), (Arabic > science), (math > science), (math > Arabic), and (science > Arabic) were used to obtain the discrepancies to identify the students who may have had a learning disability. The analysis revealed that 1443 students showed a discrepancy; hence, 23.6% of the student sample showed indicators consistent with academic learning disabilities.

Out of the 1443 students identified as showing indicators of a learning disability, 683 (47.3%) completed the consent form and took the successful intelligence test. Out of the 683 students in this subgroup showing indicators of learning disabilities, 167 were identified as gifted because their intelligence test scores were above the cut-off value of 120. This represents 24.5% of the tested subsample. Extrapolating this proportion to the full stage 1 group suggests that approximately 5.9% of students in this sample may have been twice-exceptional, that is, gifted students with learning disabilities. However, this figure assumes that the students who did not participate were broadly comparable to those who did, an assumption we cannot verify. Table 2 shows the breakdown of the prevalence rates of gifted students with learning disabilities.

The prevalence of gifted students among students with learning disabilities was explored across different age groups, genders, and program types. Table 2 presents the distribution and estimated prevalence rates of gifted students among students with learning disabilities. In primary schools, the prevalence of gifted students among students with learning disabilities varied between 11.6% for girls attending international schools (*n* = 5 out of 43) and 26.7% for boys attending local schools (*n* = 39 out of 146). In intermediate schools, the prevalence of gifted students among students with learning disabilities varied between 9.8% for girls attending local schools (*n* = 5 out of 51) and 29% for boys attending local schools (*n* = 27 out of 93). The pattern remained consistent in high schools. The prevalence of gifted students among students with learning disabilities varied between 17.5% for girls attending local schools (*n* = 7 out of 40) and 60% for boys attending international schools (*n* = 15 out of 25). These results suggest that the prevalence of giftedness among students with learning disabilities differed across age groups, genders, and school types.

### 3.3. Analysis of Student Sample by Gender

A chi-square test of independence was performed to explore the relationship between gifted students with learning disabilities and gender. Table 3 presents the crosstabulation of the two variables. The relationship between these variables was significant: χ^2^(1, *N* = 675) = 18.77 (*p* < .001). Boys with learning disabilities appeared more likely than girls to be identified as gifted (30.4% (95% CI: [26.0%, 34.8%]) vs. 15.6% (95% CI: [11.1%, 20.0%])), suggesting a small effect size (Cramer’s *V* = 0.17).

### 3.4. Analysis of Student Sample by Age Groups

A chi-square test of independence was performed to explore the relationship between gifted students with learning disabilities and different age groups. Table 4 presents the crosstabulation of the two variables. The relationship between these variables was significant: χ^2^(2, *N* = 675) = 19.22 (*p* < .001). Students with learning disabilities in high school appeared more likely to be identified as gifted (38.4%; 95% CI: [30.3%, 46.5%]) than those in intermediate (24.2%; 95% CI: [18.6%, 29.8%]) or primary school (19.1%; 95% CI: [14.8%, 23.5%]), suggesting a small effect size (Cramer’s *V* = 0.17).

### 3.5. Analysis of Student Sample by Program Type

A chi-square test of independence was performed to explore the relationship between gifted students with learning disabilities and program type. Table 5 presents the crosstabulation of the two variables. The relationship between these variables was not significant: χ^2^(1, *N* = 675) = 0.04 (*p* = .840); Cramer’s *V* = 0.01. The prevalence of gifted students with learning disabilities was similar across local (24.5%; 95% CI: [20.6%, 28.4%]) and international programs (25.2%; 95% CI: [19.4%, 31.1%]).

### 3.6. Analysis of Student Sample by Learning Disabilities and School Subjects

A chi-square test of independence was performed to explore the relationship between gifted students with learning disabilities and specific academic subjects. In other words, we examined whether a particular school subject (i.e., math, Arabic, science, or multiple academic subjects) was more likely to be associated with being a gifted student with a learning disability. Table 6 presents the crosstabulation of the two variables. The relationship between these variables was significant: χ^2^(3, *N* = 582) = 54.78 (*p* < .001). Students with disabilities in Arabic (42.8%; 95% CI: [35.1%, 50.5%]) and science (27.8%; 95% CI: [17.4%, 38.1%]) appeared more likely to be identified as gifted than those with disabilities in mathematics (21.9%; 95% CI: [16.3%, 27.5%]) or multiple subjects (6.4%; 95% CI: [2.3%, 10.4%]), suggesting a large effect size (Cramer’s *V* = 0.31).

## 4. Discussion

The observation and diagnosis of twice-exceptional students is historically challenging due to the interplay between cognitive “strengths” and “weaknesses,” referred to as the “masking effect.” In addition, the challenge of masking their characteristics (McEachern & Bornot, 2001) is further complicated by a lack of agreement on the best approach to identifying these students (Maddocks, 2018). As a result, it has become difficult to estimate their prevalence (Foley-Nicpon et al., 2013). However, before advocating for educational policies ensuring that students with learning disabilities receive the necessary resources to support their giftedness, it is important to first assess the prevalence of these students among our student population.

Hence, this study aimed to explore the prevalence of twice-exceptional students in Saudi Arabia, particularly focusing on gifted students with learning disabilities. The investigation involved a sample of 6875 students drawn from four schools in Riyadh, spanning from first to twelfth grade. The analysis sought to explore the association between gifted students with learning disabilities and various academic subjects, age groups, and program types. Researchers utilized academic subject discrepancy as a screening indicator for learning disabilities, a method commonly employed in prior studies to identify learning disabilities in twice-exceptional students (McCallum et al., 2013; McClurg et al., 2021) and an IQ of 120 as an indicator of giftedness, a criterion also frequently used in previous research to identify giftedness in twice-exceptional students (Al-Hroub & Whitebread, 2008; Maddocks, 2020).

Stage 1 was designed as a screening procedure rather than a diagnostic one. The discrepancies in academic performance observed may not always indicate an underlying learning disability in the clinical sense, so the estimate of 5.9% should be understood as a preliminary indication of students whose academic profiles are consistent with twice-exceptionality within this sample.

The initial findings of this study reveal that approximately 5.9% of the sampled population may qualify as twice-exceptional students, aligning with previous research in the field. Baum et al. (2017) reported that approximately 6% of the student population displayed traits of being twice exceptional, while Reis and McCoach (2000) reported a prevalence rate ranging from 5% to 7% for gifted students with learning disabilities. Moreover, Maddocks (2018) identified 2.6% of the total sample as potentially gifted students with learning disabilities, whereas in their subsequent study in 2018, this figure rose to 15.6% of the total sample. The variation in prevalence rates across previous studies may stem from differences in the definitions of giftedness and learning disabilities, as well as variations in their prevalence rates (Gagné et al., 1993; Renzulli & Renzulli, 2010; Ronksley-Pavia, 2020). Overall, our findings suggest that the estimated prevalence of gifted students with learning disabilities in this study mirrors that of prior research, highlighting the substantial proportion of students exhibiting both giftedness and learning disabilities. This underscores the importance of identifying and supporting these students to address their unique educational needs. Furthermore, extending the findings of prior investigations, this study suggests that the prevalence of gifted students among those with learning disabilities may vary across genders, age groups, and program types.

Additionally, this study suggests that males were more likely than females to be identified as gifted students with learning disabilities, consistent with findings from a systematic review of non-cognitive characteristics of such students, where males predominated among the studies included (Beckmann & Minnaert, 2018). This gender difference may be explained by the higher representation of males among students diagnosed with disabilities, thus leading to an elevated rate of diagnoses of gifted students with learning disabilities among males (Johnson, 2017; Morgan, 1979; Nass, 1993).

Age emerged as a potentially significant factor influencing the prevalence rate, with this study’s results suggesting a rise in the overall average prevalence rate as students advanced through the grades. This finding aligns with Maddocks’s (2020) research, which found a prevalence of 15% for gifted students with learning disabilities among grade 12 students. This trend can be elucidated from the existing literature on twice-exceptional students, which suggests that their challenges become more pronounced with age. As academic difficulties in their areas of weakness intensify over time, there is often a decline in academic performance and an increasingly evident gap between grades in academic courses (Hamzić & Bećirović, 2021; Silver, 2008).

Furthermore, the variable of language of study in the program (Arabic as the native language in Saudi Arabia and English as a second foreign language) did not significantly impact the prevalence of gifted students with learning disabilities. This lack of disparity may be attributed to the administration of the IQ test in both Arabic and English, allowing students to opt for the language in which they are most proficient. Consequently, students studying in Arabic may opt for the IQ test in their primary language, while those in English-language programs may choose to take it in English. Given that all students are native Arabic speakers, regardless of program type, discrepancies observed in Arabic performance are unlikely to reflect a language proficiency gap. Instead, they may genuinely reflect underlying learning difficulties, which supports the validity of including Arabic as a screening subject across both programs.

Regarding the impact of disability type on the mean estimated prevalence of gifted students with learning disabilities, the investigation suggested a higher likelihood of identifying giftedness among students with specific language (i.e., Arabic) and science disabilities compared with those with mathematics disabilities. This finding is broadly consistent with previous scholarly inquiries, exemplified by the systematic review conducted by Beckmann and Minnaert (2018), which indicated a predominant focus on gifted students with language-related challenges in the existing literature. One possible explanation is that language difficulties are among the most common forms of learning difficulty (Semrud-Clikeman & Ellison, 2009). These language difficulties can profoundly influence scientific subjects, given that many scientific tasks necessitate adept language skills for their comprehension and resolution.

## 5. Limitations and Future Research

This study provides valuable exploratory insights into the prevalence rates of gifted students with learning disabilities, offering detailed estimates stratified by gender, academic year, language of instruction, and type of learning disability. Nevertheless, it has some limitations, such as this study’s methodology, particularly concerning the detection of gifted students with learning disabilities. The use of an IQ threshold of 120 as an indicator of giftedness and the utilization of achievement variance as a marker for learning disabilities may yield varied prevalence rates compared with alternative methodologies, such as curriculum-based assessment (McCallum et al., 2013). Furthermore, given that the screening process identifies learning difficulties before assessing giftedness, students whose abilities mask their difficulties will not demonstrate sufficient academic discrepancy to progress to stage 2. This subgroup is the hardest to detect, and the estimate of 5.9% is likely an undercount. Future studies would benefit from assessing giftedness and learning difficulties simultaneously rather than sequentially.

Another major constraint concerns this study’s sample composition. Despite encompassing four schools in Riyadh and a sample size of around 7000 students, all participants were drawn from private educational institutions. Consequently, the economic and cultural backgrounds of these students may diverge from those of the broader student populace in Riyadh. Given that previous research has identified economic and cultural status as a determinant influencing both giftedness (e.g., Hodges & Gentry, 2021; McBee, 2006) and learning difficulties (e.g., Fields, 1999; Shifrer et al., 2011), future investigations should comprise students from public schools as well. Moreover, it is highly recommended that researchers examine whether disparities exist in the prevalence of gifted students with learning disabilities across varying economic and cultural contexts, ideally using designs that also address the consent attrition challenges discussed below. This study was conducted in Riyadh; replication in other Saudi regions and international contexts would help establish the findings’ generalizability. Prevalence estimates for twice-exceptional students vary across countries, depending on the definitions and identification procedures used. This study provides an initial contribution to the wider discussion, rather than providing a definitive estimate.

Furthermore, relying on grade-based discrepancy as a first-stage screening criterion requires careful consideration. Academic grades can vary remarkably depending on the context, including differences in instructional quality, student motivation, and teacher assessment practices. Moreover, no exclusion criteria were applied, meaning some of the observed discrepancies may have been due to factors such as sensory impairment, socioeconomic disadvantage, or inadequate instruction rather than a genuine learning disability. Students identified in stage 1 should, therefore, be understood as demonstrating significant academic performance discrepancies rather than possessing clinically confirmed learning disabilities, and prevalence estimates should be interpreted accordingly.

Additionally, giftedness in this study was identified solely via IQ scores. Though this method agrees with standard procedures in large-scale research (Carman, 2013), it does not capture the full definition of giftedness, which includes various factors, such as creativity and socio-emotional traits. Therefore, future studies would benefit from incorporating broader, multidimensional assessments.

A further limitation of the study is the presence of missing data at two stages. Firstly, 11% of the initial sample was excluded due to incomplete grade records. If the excluded students disproportionately comprised those with greater academic difficulties, the stage 1 identification rate may be slightly underestimated. Secondly, and more substantially, fewer than half of the students identified in stage 1 completed the intelligence assessment. As no additional information was available about either group beyond their school records, whether they differed systematically from those who were included cannot be determined. Both sources of attrition introduced uncertainty into the 5.9% conditional estimate and should be considered when interpreting the findings. Future research would benefit from retaining participants across both stages and collecting basic background data on all students identified during the screening process.

## 6. Conclusions

Identifying twice-exceptional students is complicated by the complexity of discerning the coexistence of high abilities alongside learning, emotional, physical, sensory, and/or developmental disabilities (Foley-Nicpon et al., 2011). Nonetheless, it remains essential to conduct studies exploring the prevalence of this demographic among students. These challenges have fueled ongoing discussions regarding the distinctive cognitive profiles of twice-exceptional students compared with those who are solely gifted or disabled. Despite this complexity, undertaking such studies holds significance, as understanding the proportions of this population can yield numerous theoretical and practical implications extending beyond local contexts to the countries conducting such investigations.

At the national level, this study suggests that the prevalence of twice-exceptional students within this private school sample may be higher than previously reported in the Saudi context. A prior study conducted in the Kingdom, albeit with a limited sample size of 244 students, reported a prevalence of approximately 3% (Bakhiet & Essa, 2012). However, this investigation, using a larger and more diverse sample, yielded a conditional estimate of 5.9%. This points to a meaningful group of underserved students. Consequently, the findings highlight the value of developing targeted identification procedures and support programs for this population. A further investigation in this area is warranted before broader policy recommendations can be made.

Our study offers significant broader theoretical implications by addressing a gap in prior research. To the best of our knowledge, no previous study has undertaken such a large-scale exploratory examination of the prevalence rates of gifted students with learning disabilities. The prior literature has underscored the necessity of exploring disparities in prevalence rates across age groups and specific types of difficulties (Beckmann & Minnaert, 2018). Thus, our study contributes to the theoretical discourse by exploring variations in the prevalence rates of gifted students with learning disabilities among students, delineated by gender, age, and specific difficulty types. This highlights the nuanced characteristics of this demographic across these variables. Consequently, this finding underscores the potential value of future studies adopting a more comprehensive approach, aiming to identify suitable identification methods and educational interventions tailored to each subgroup within this population.

In terms of practical implications, our findings suggest the importance of raising awareness among teachers and parents regarding the substantial number of students with learning disabilities who also exhibit giftedness. For instance, our study suggests that 24.5% of students with learning disabilities may demonstrate gifted characteristics. However, many educators and parents may overlook indicators of giftedness in students with learning disabilities, potentially influenced by stereotypes suggesting that gifted students must excel in all subjects (Baudson & Preckel, 2016; Callahan, 2001). Consequently, it would be valuable to inform teachers and parents that a discernible gap in a student’s abilities is a crucial indicator warranting attention. Such students should be referred for further assessment to ascertain whether their difficulties mask underlying high abilities. This awareness can facilitate the identification and appropriate support of these students, ultimately fostering their academic and personal development.

A pertinent implication arising from our study is the potential need to revise the procedures for identifying gifted students and the instruments employed in this process. Currently, many countries use nomination as the initial step in identification (e.g., the United States (National Association for Gifted Children, 2025); Switzerland, Germany, and France (Sękowski & Łubianka, 2015); Hong Kong (Chan, 2000); and Saudi Arabia (A. Aljughaiman et al., 2016)). Previous studies suggested a method based on academic achievement (Barber & Torney-Purta, 2008; Hernández-Torrano et al., 2013; Kornmann et al., 2015; Siegle & Powell, 2004). However, this approach may inadvertently exclude a significant proportion of gifted students, particularly those with low academic achievement. Furthermore, many identification instruments heavily prioritize language proficiency, potentially excluding gifted students with learning disabilities, particularly in language-related areas. A subgroup of our study constituted a significant portion of gifted students with learning disabilities.

To conclude, our study suggests that gifted students with learning disabilities constitute a substantial segment of the student population, based on both the present exploratory findings and prior research on this subject. Therefore, the preliminary findings of this study suggest that more attention should be given to this population in educational planning, although this needs to be replicated with more representative samples. Such efforts would help ensure that adequate resources and qualified personnel are in place to support these students’ unique strengths and challenges.

## Figures and Tables

**Table 1:** Psychometric properties of students’ grades.

Variable	α	M	SD	Range	Skewness	Kurtosis
Arabic grade	0.79	79.84	19.066	96	–1.02	0.40
Mathematics grade	0.83	72.74	22.670	95	−0.60	−0.67
Science grade	0.83	74.72	20.951	95	−0.67	−0.39
Successful intelligence test	0.79	108.71	16.07	70	−0.64	−0.36

**Table 2:** Breakdown of prevalence rates of gifted students showing indicators of learning disabilities.

Age Group	Gender	Program Type	Total Number	Students with Indicators of Learning Disabilities	Students Assessed for Giftedness (Stage 2)	Students with Learning Disabilities and Gifted	Prevalence of Gifted Students Among Those with Learning Disabilities (%)
Primary	Boys	Local	1174	322	146	39	26.7%
Primary	Boys	International	405	74	37	5	13.5%
Primary	Girls	Local	740	236	75	11	14.6%
Primary	Girls	International	333	54	43	5	11.6%
Intermediate	Boys	Local	939	180	93	27	29.0%
Intermediate	Boys	International	305	77	60	17	28.3%
Intermediate	Girls	Local	593	115	51	5	9.8%
Intermediate	Girls	International	218	45	21	5	23.8%
High school	Boys	Local	675	166	58	24	41.3%
High school	Boys	International	194	48	25	15	60.0%
High school	Girls	Local	406	101	40	7	17.5%
High school	Girls	International	138	25	15	7	46.7%

*Note.* “Students assessed for giftedness (Stage 2)” represents the number of students showing indicators of learning disabilities who proceeded to the second screening stage and received an IQ assessment. The prevalence rate was calculated as the proportion of students scoring IQ ≥ 120 among those assessed.

**Table 3:** The crosstabulation of gifted students with learning disabilities and gender.

		Gender		Total
		Girls	Boys	
Gifted with learning disabilities	No	217	291	508
	Yes	40	127	167
Total		257	418	675

**Table 4:** The crosstabulation of gifted students with learning disabilities and age groups.

		Age Group			Total
		Primary	Intermediate	High School	
Gifted with learning disabilities	No	254	169	85	508
	Yes	60	54	53	167
Total		314	223	138	675

**Table 5:** The crosstabulation of gifted students with learning disabilities and program type.

		Program Type		Total
		Local	International	
Gifted with learning disabilities	No	348	160	508
	Yes	113	54	167
Total		461	214	675

**Table 6:** The crosstabulation of gifted students with learning disabilities and specific academic subjects.

		Academic Subject				Total
		Math	Arabic	Science	Multiple Academic Subjects	
Gifted with learning disabilities	No	164	91	52	132	439
	Yes	46	68	20	9	143
Total		210	159	72	141	582

## Data Availability

The data underlying this study are available from the corresponding author on request; however, they are not publicly shared due to confidentiality and privacy considerations.
